# Stromal Senp1 promotes mouse early folliculogenesis by regulating BMP4 expression

**DOI:** 10.1186/s13578-017-0163-5

**Published:** 2017-07-25

**Authors:** Shu Tan, Boya Feng, Mingzhu Yin, Huanjiao Jenny Zhou, Ge Lou, Weidong Ji, Yonghao Li, Wang Min

**Affiliations:** 1 Department of Pathology and the Vascular Biology and Therapeutics Program, Yale University School of Medicine, New Haven, CT 06519 USA; 2 Center for Translational Medicine, The First Affiliated Hospital, Sun Yat-sen University, Guangzhou, 510080 China; 3 Department of Gynecology Oncology, The Tumor Affiliated Hospital of Harbin Medical University, Harbin, China; 4 State Key Laboratory of Ophthalmology, Zhongshan Ophthalmic Center, Sun Yat-sen University, Guangzhou, China

## Abstract

**Background:**

Mammalian folliculogenesis, maturation of the ovarian follicles, require both growth factors derived from oocyte and surrounding cells, including stromal cells. However, the mechanism by which stromal cells and derived factors regulate oocyte development remains unclear.

**Results:**

We observed that SENP1, a small ubiquitin-related modifier (SUMO)-specific isopeptidase, was expressed in sm22α-positive stromal cells of mouse ovary. The sm22α-positive stromal cells tightly associated with follicle maturation. By using the sm22α-specific Cre system, we show that mice with a stromal cell-specific deletion of SENP1 exhibit attenuated stroma-follicle association, delayed oocyte growth and follicle maturation with reduced follicle number and size at early oocyte development, leading to premature ovarian failure at late stages of ovulating life. Mechanistic studies suggest that stromal SENP1 deficiency induces down-regulation of BMP4 in stromal cells concomitant with decreased expression of BMP4 receptor BMPR1b and BMPR2 on oocytes.

**Conclusions:**

Our data support that protein SUMOylation-regulating enzyme SENP1 plays a critical role in early ovarian follicle development by regulating gene expression of BMP4 in stroma and stroma-oocyte communication.

**Electronic supplementary material:**

The online version of this article (doi:10.1186/s13578-017-0163-5) contains supplementary material, which is available to authorized users.

## Background

Folliculogenesis is the maturation of the ovarian follicle, a densely packed shell of somatic cells that contains an immature oocyte. Mouse ovarian development can be divided into several steps: (1) germ cysts breakdown and primordial follicle formation, (2) primordial follicle activation and development to advanced-stage follicles, (3) ovulation or apoptosis. Within the ovary in mice, primordial germ cells arrested in urogenital ridges to undergo mitosis, results in oocytes cluster, which subsequently to form germ cell clusters [1–6]. Following the programmed breakdown of germ cell cysts shortly after birth, only one-third of individual oocytes enveloped by a layer of flat somatic pregranulosa cells, which eventually become primordial follicles [7–9]. With continuous loss and apoptosis of oocytes after birth by unknown mechanisms, selected primordial follicles recruit a single layer of cuboidal granulosa cells with oocytes grow inside to form primary follicles, which in turn mature into advanced follicles. It is known now that the oocytes numbers in adult are tightly associated with the finite primordial follicle reservoir. Moreover, the breakdown of germ cell clusters, the cell proliferation in primordial follicle formation and the transition from primordial follicle into primary follicle is critical for subsequent folliculogenesis, i.e., progression of a number of small primordial follicles into large preovulatory follicles [7–9]. Recent studies suggest that folliculogenesis requires both oocyte intrinsic self-organization and complex communications with surrounding somatic cells, involving multiple autocrine and paracrine signaling pathways [10–14]. In particular, specific cytokines and growth factors derived from stromal cells are required for activation of primordial follicle and maturation of oocytes [7–9, 15].

The small ubiquitin-like modifier (SUMO) can be covalently attached to a large number of proteins through formation of isopeptide bonds with specific lysine residues of target proteins [16]. SUMO (SUMO1, 2 and 3) with SUMO1 more broad specificity [17], is covalently attached to substrate proteins via an isopeptide bond between a C-terminal glycine and a lysine residue in the substrate. A consensus SUMO acceptor site has been identified consisting of the sequence ØKXE (Ø is a large hydrophobic amino acid and K is the site of SUMO conjugation). The consequence of SUMOylation on protein function is substrate specific, regulating protein stabilization, localization, protein–protein or protein–DNA interactions, and/or biochemical activities. SUMOylation is a dynamic process that is mediated by activating (E1), conjugating (E2), and ligating (E3) enzymes and is readily reversed by a family of SUMO-specific proteases with 6 members [18]. SENP1 is a protease that appears to be localized in several compartments and deconjugates a large number of SUMOylated proteins [18–20]. Recently, protein post-translational modification SUMOylation has been reported to play an important role in germ cell function, especially in mammalian meiosis [21–23]. Several studies have characterized expression of SUMO-1 and SUMO-2/3 in oocytes. While SUMO-2/3 proteins are localized in nucleoplasm, SUMO-1 is concentrated at spindle organization and chromosome in transcriptionally active oocytes with little location on nuclear membrane in quiescent oocytes. Moreover, this specific localization of SUMO-1 plays a critical role during oocytes maturation [21–23]. It has also been reported that differential localization of SENP1 regulate SUMOylation in a temporal and spatial fashion along the oocyte meiosis procession [24, 25]. However, little is known about role of protein SUMOylation in stromal cells surrounding oocyte in regulating follicle development and oocyte maturation is unclear. Here, we show that stromal deletion of SENP1 in mice, by increasing cellular SUMOylation and decreasing BMP4 expression, retards oocytes growth and follicle formation at early developmental stage.

## Results

### SM22α-positive stromal cells surround germ cells and oocytes

**Fig. 1:**
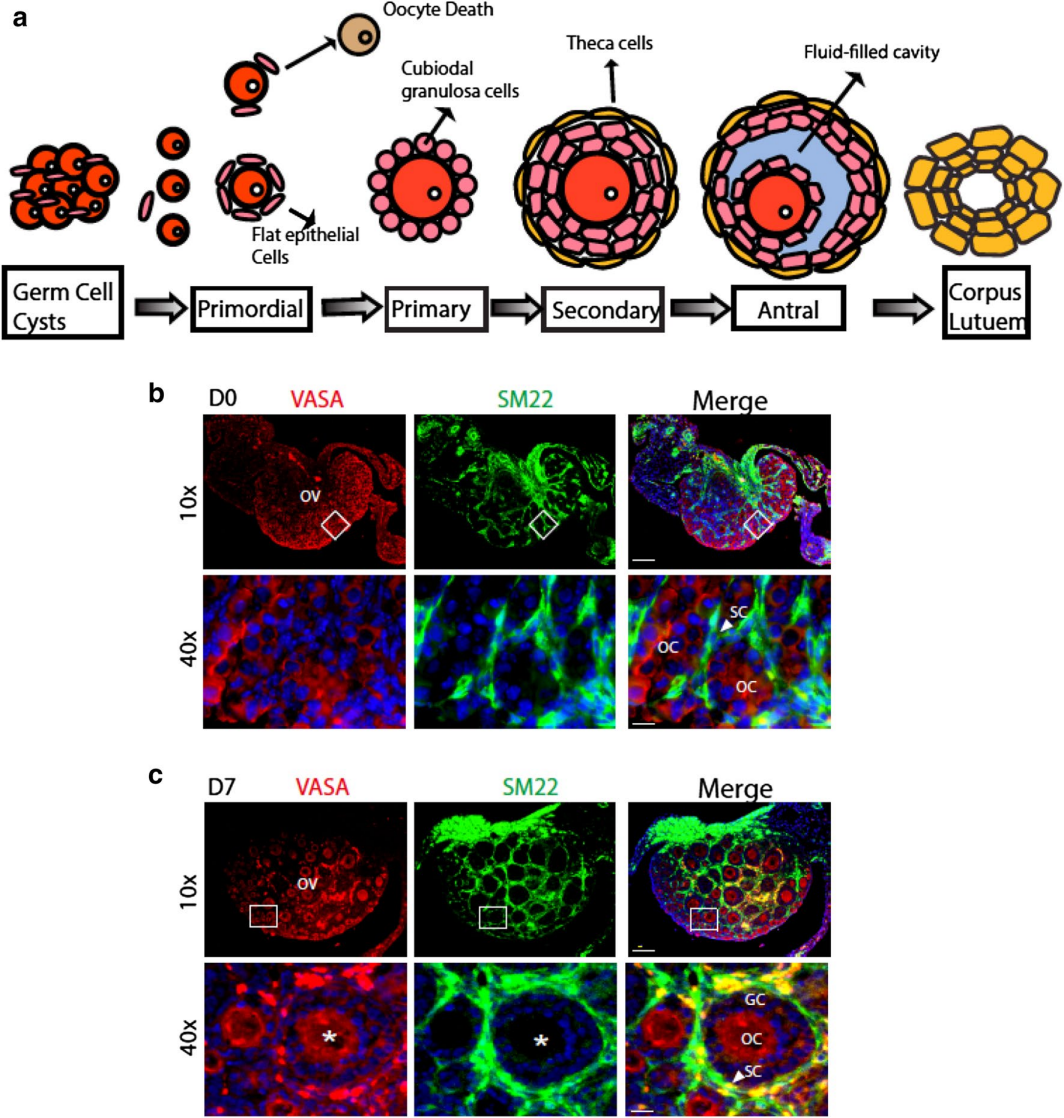
Identification of SM22α-positive stromal cells in mouse ovary development. **a** Diagram for mammalian oocyte development within ovarian follicles. Shortly after birth, mouse germ cell cysts undergo programmed breakdown while single oocyte survive to form primordial follicles. As follicles grow, granulosa cells keep proliferating (one-layer to multi-layer) until fluid-filled cavities (the antrum) appear between the layers of somatic cells at later stage of follicle development. At the same time, stromal cells (not shown in the diagram) develop into mature stage to surround the layers of theca/granulosa cells. **b**, **c** SM22α stromal cells surround oocytes. DDX4 (DEAD-box helicase 4; also known as VASA) and stromal cell marker SM22α were used co-immunofluorescence in ovaries of newborn (D0; **b**) and 7-day-old (**c**; D7) WT mice. In newborn ovaries, stromal cells (*green*) were arranged in strips within the interval of germ cells (*red*). Stromal cells become mature and envelope layers of granulosa cells in advanced follicles. *Scale bars* 60 μm in ×10 images; 15 μm in ×40 images. *OV* ovary, *OC* oocyte, *GC* granulosa cell, *SC* stromal cell. *Asterisks* indicate the same oocyte in the merged panel

Classical studies identified that stromal/somatic cells interact closely with germ cells as early as 14.5 dpc (day past coitum) in mouse ovarian development [1–3]. Stromal cells and stromal progenitor cell insert between closely associated germ cells followed by germ cysts breakdown as part of the progress results in primordial follicle formation. Moreover, these stromal progenitor cells undergo differentiation and maturation into various cell types, including flat epithelial cells, cuboidal granulosa cells and theca cells that support oocyte maturation (Fig. 1a). It is reported that the stromal cells secret growth factors critical for oocyte maturation. However, the nature and function of these stromal cells have not been investigated. By performing co-immunofluorescence staining with germ cell marker VASA protein (also named DDX4/MVH), we initially observed that stromal cells express smooth muscle cell progenitor marker SM22α (Fig. 1b, c). VASA expression could be detected as early as primordial germ cells (PGCs) and was expressed throughout oocyte development stages, including primary, secondary and mature oocytes. VASA^+^ primordial and primary follicles were juxtaposed with SM22α^+^ cells at D0 (Fig. 1b), and subsequently the mature oocytes along with granulosa cells were tightly surrounded by SM22α^+^ stromal cells (Fig. 1c for D7).

### Stromal SENP1 deletion accelerates premature ovarian failure at late stages of ovulating life

**Fig. 2:**
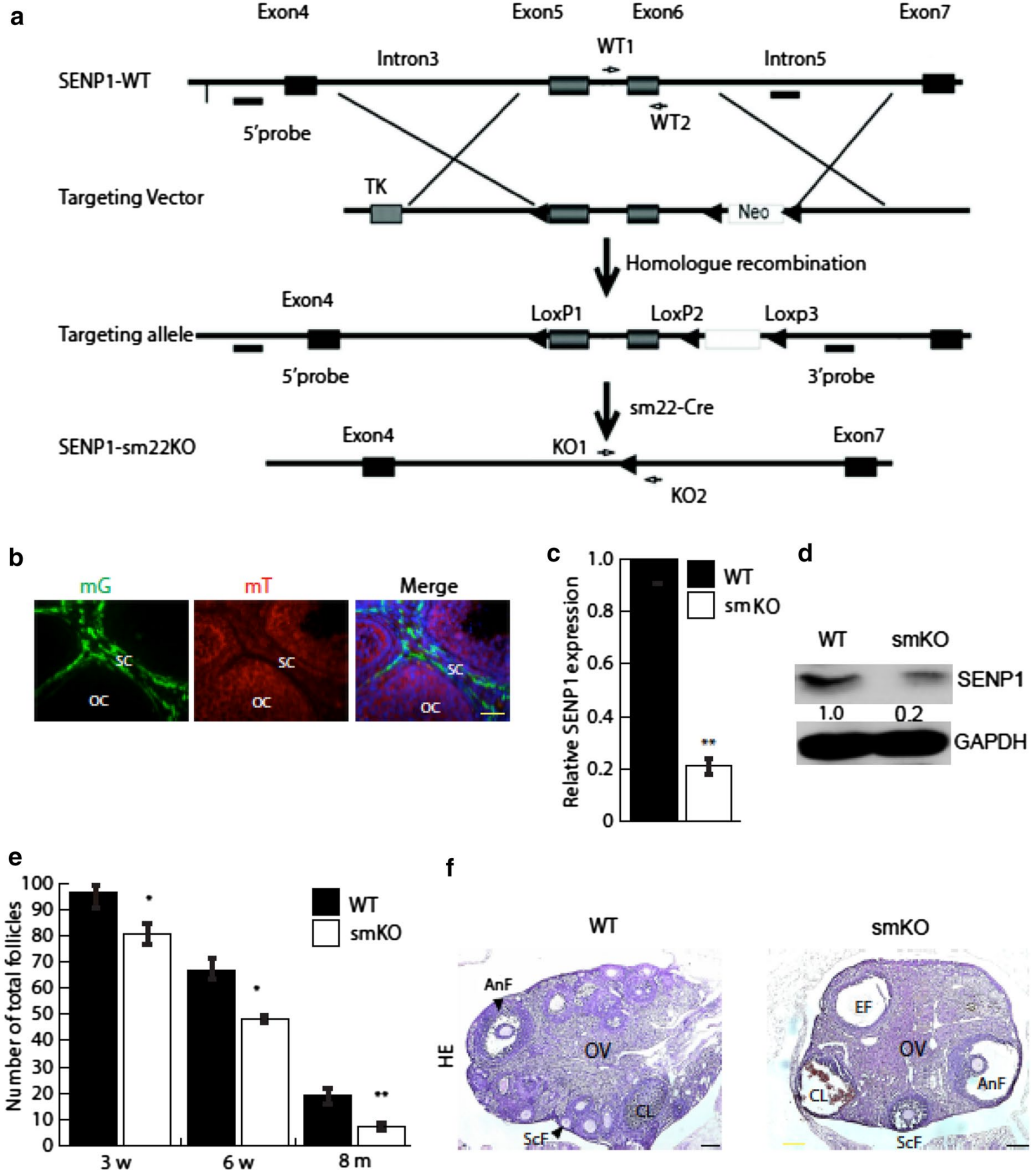
Premature ovarian failure appeared in later stage of SENP1-smKO mice ovulating life. **a** Stromal deletion of SENP1 with SM22α-Cre. SENP1^lox/lox^ mice were generated based on homologous recombination. SENP1^lox/lox^ mice were mated with SM22α-Cre to obtain stromal-specific SENP1-deficient (SENP1-smKO) mice. 5′ and 3′ probes were used for Southern blot. Primers WT1/2 and KO1/2 were used for genotyping. **b** SM22α cells are in ovarian stroma. The SM22α-Cre deleter mice were mated with mice expressing a genetic Cre reporter (ROSA-26Sor^tm4(ACTB−tdTomato, EGFP)Luo^/J). Stromal cells show GFP-positive, suggesting these cells are SM22α lineage. **c**, **d** SENP1 deletion in SM22α stromal cells. SM22α-positive stromal cells were isolated from ovaries of SENP1^lox/lox^ (WT) and SENP1-smKO female mice. SENP1 expression was detected by qRT-PCR (**b**) and Western blotting (**c**). Normalized mRNA and protein levels (with GADPH) are presented as fold changes by taking WT group as 1.0. Data are shown as mean ± SEM, n = 3, female. **e** Stromal SENP1 deletion reduces follicle numbers. Number of total follicle in ovaries of WT and SENP1-smKO mice at ages of 3, 6 weeks and 8 months. Data are presented as mean ± SEM, n = 5, *P < 0.05, **P < 0.01. **f** Premature ovarian failure in aged SENP1-smKO mice. Representative Hematoxylin and Eosin staining of ovaries from 9-month-old WT and SENP1-smKO (n = 5). *Scale bars* 160 μm. *OV* ovary, *ScF* secondary follicle, *AnF* antral follicle, *CL* corpus lutuem

Based on the role of protein SUMOylation in germ cell function, we reasoned that a specific deletion of deSUMOylase SENP1 in SM22α-positive stromal cells would affect follicle formation. To test our hypothesis, we created mice with an SM22α-specific SENP1 deletion by mating SENP1^f/fl^ with SM22α-Cre mice (Fig. 2a). We examined the SM22α lineage in the ovary by mating SM22α-Cre with mG/mT reporter mice [26], and results indicated that SM22α cells were located within ovarian stroma (Fig. 2b). SENP1 deletion in SM22α-positive cells was verified by qRT-PCR and Western blotting by isolating SM22α-positive cells from ovaries of WT (SENP1^f/fl^) and SENP1-smKO (SM22α-Cre; SENP1^f/fl^) (Fig. 2c, d). To explore how protein SUMOylation affects the reproductive life in mouse, ovaries sections from WT and SENP1-smKO mice at various ages (3–9 weeks) were examined by H&E staining and the numbers of follicles at various stages were quantified (Fig. 2e). The number of follicles in ovaries from SENP1-smKO mice was significantly reduced compared to WT mice at all ages (3 and 6 weeks till 8 months), with more severe reduction at older ages (>8 month older) (Fig. 2e, f). At 8 months postpartum, different stages developing follicles (primary, secondary and advanced stages) and corpus lutuem were detected in WT ovaries. SENP1-smKO ovaries contained very few follicles and much more empty follicles without oocytes, implicating that ovaries were degenerated.

### Stromal SENP1 deletion attenuates oocyte growth and follicle formation

**Fig. 3:**
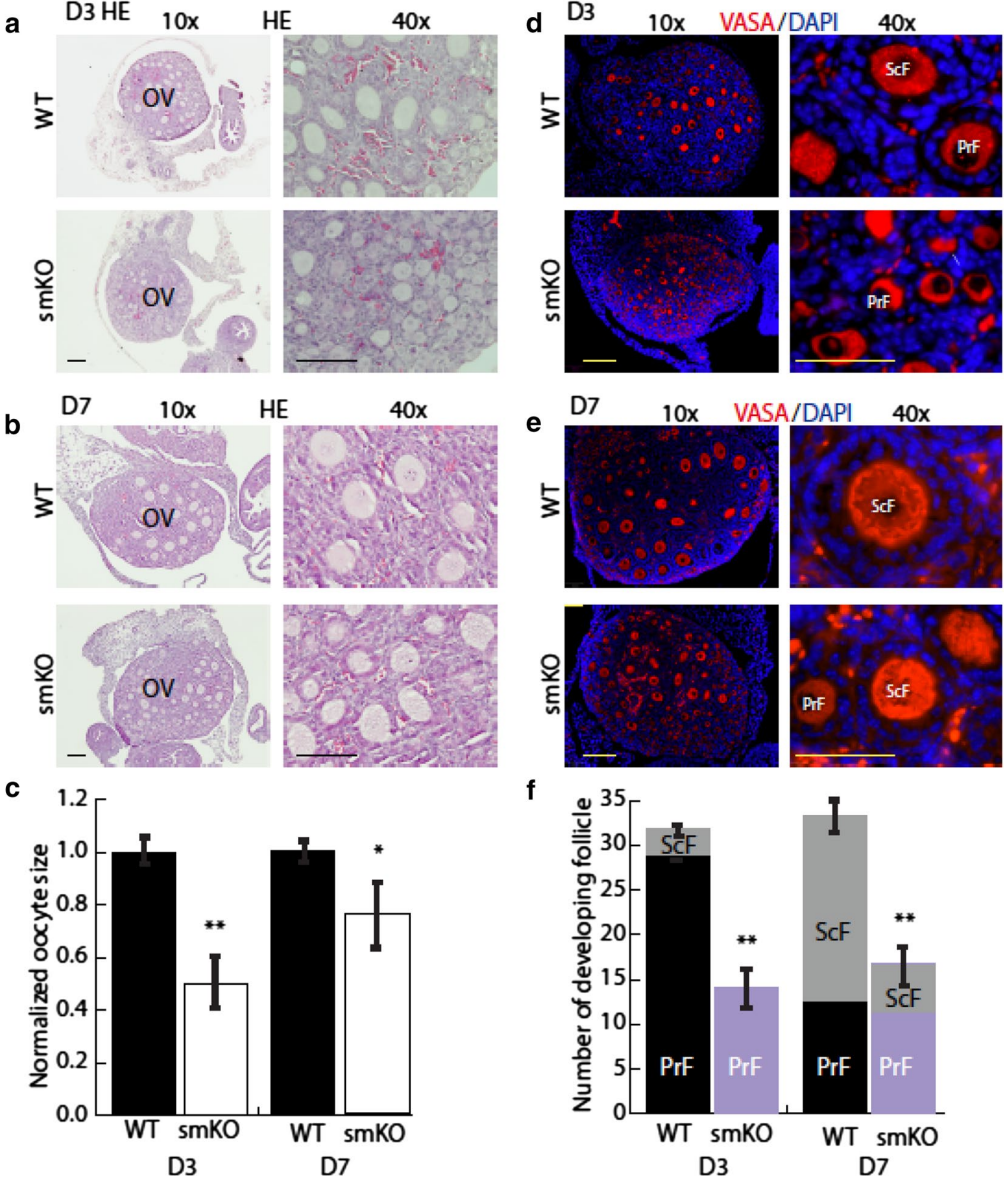
Stromal SENP1 deletion delays early follicle development in neonatal mouse. **a**–**c** Stromal SENP1 deletion reduces follicle size and number in neonatal mice. Hematoxylin and Eosin staining of ovaries from WT and SENP-smKO mice at postnatal day 3 (**a**) and day 7 (**b**). *Scale bars* 160 μm. Sizes of primary and secondary follicles in ovaries of WT and SENP1-smKO mice are quantified (**c**). Data are presented as mean ± SEM, n = 5, *P < 0.05, **P < 0.01. **d**–**f** Stromal SENP1 deletion delays early folliculogenesis in neonatal mice. VASA immunofluorescence staining of ovaries from WT and SENP-smKO mice at postnatal day 3 (**d**) and day 7 (**e**). *Scale bars* 60 μm in ×10 images; 15 μm in ×40 images. Number of primary and secondary follicles are quantified (**f**). Data are presented as mean ± SEM, n = 5, **P < 0.01. *OV* ovary, *PrF* primary follicle, *ScF* secondary follicle

The premature ovarian failure of SENP1-smKO mice at 8 months old prompted us to examine early stages of ovarian development, including activation of primordial, primary and secondary follicles. Germ cell cysts were defined as two or more oocytes that were not individually separated by stromal cells. Primordial follicles were defined as small oocytes (<20 μm) surrounded by flat epithelial cells; primary follicles were defined as having larger oocytes (>20 μm) surrounded by a single layer of cuboidal and proliferative granulosa cells; secondary follicles were defined as larger oocytes surrounded by two or more layers of granulosa cells (see Fig. 1a) [1–3]. On basis of previous studies in mouse ovarian development, primary-stage follicles and secondary-stage follicles are formed on postnatal day 3–7 [1–6]. We examined oocytes growth and follicle development in WT and SENP1-smKO pups on day 3–7 by H&E staining and VASA immunostaining. H&E staining suggested that oocytes in WT were evenly distributed through ovary with uniform sizes on day 3 and day 7. Oocytes in SENP1-smKO ovaries were much smaller with variable sizes on day 3 and even on day 7 (Fig. 3a, b with quantifications in Fig. 3c). In contrast, mice with a SENP1 deletion in vascular endothelial cells (SENP1-ecKO) did not significantly alter ovarian development and oocyte maturation compared to WT ovaries (Additional file 1: Figure S1A–C). These data suggest a specific role for SENP1 in SM22α-positive stromal cells. SENP1 ASA staining indicated that WT ovaries on postnatal day 3 contained a majority of primary follicles which rapidly matured into secondary follicles on day 7. However, SENP1-smKO oocytes were smaller sizes with various stages of oocytes, including germ cell cysts (GCC), primordial follicles (PrmdF), primary follicles (PrF) and secondary follicles (ScF) (Fig. 3d). By postnatal day 7, WT oocytes were at a stage of secondary follicle but most ovaries of SENP1-smKO mice were still at an early stage of primary follicle with smaller sizes (Fig. 3e). The number of developing follicles (both primary follicle and secondary follicles) was significantly lower in the SENP1-smKOs on both D3 and D7 (Fig. 3f). These results suggest that SENP1-smKO mice exhibit delayed oocyte growth and follicle development from the primordial follicle activation to advanced stages. We cannot exclude the possibility that folliculogenesis in SENP1-smKO mice might be arrested at even earlier stage, Cyst or primordial stage, or drop out of the developmental process at very early stage.

### SM22α stromal cells are disorganized with altered expression of BMPs in ovaries of SENP1-smKO mouse

**Fig. 4:**
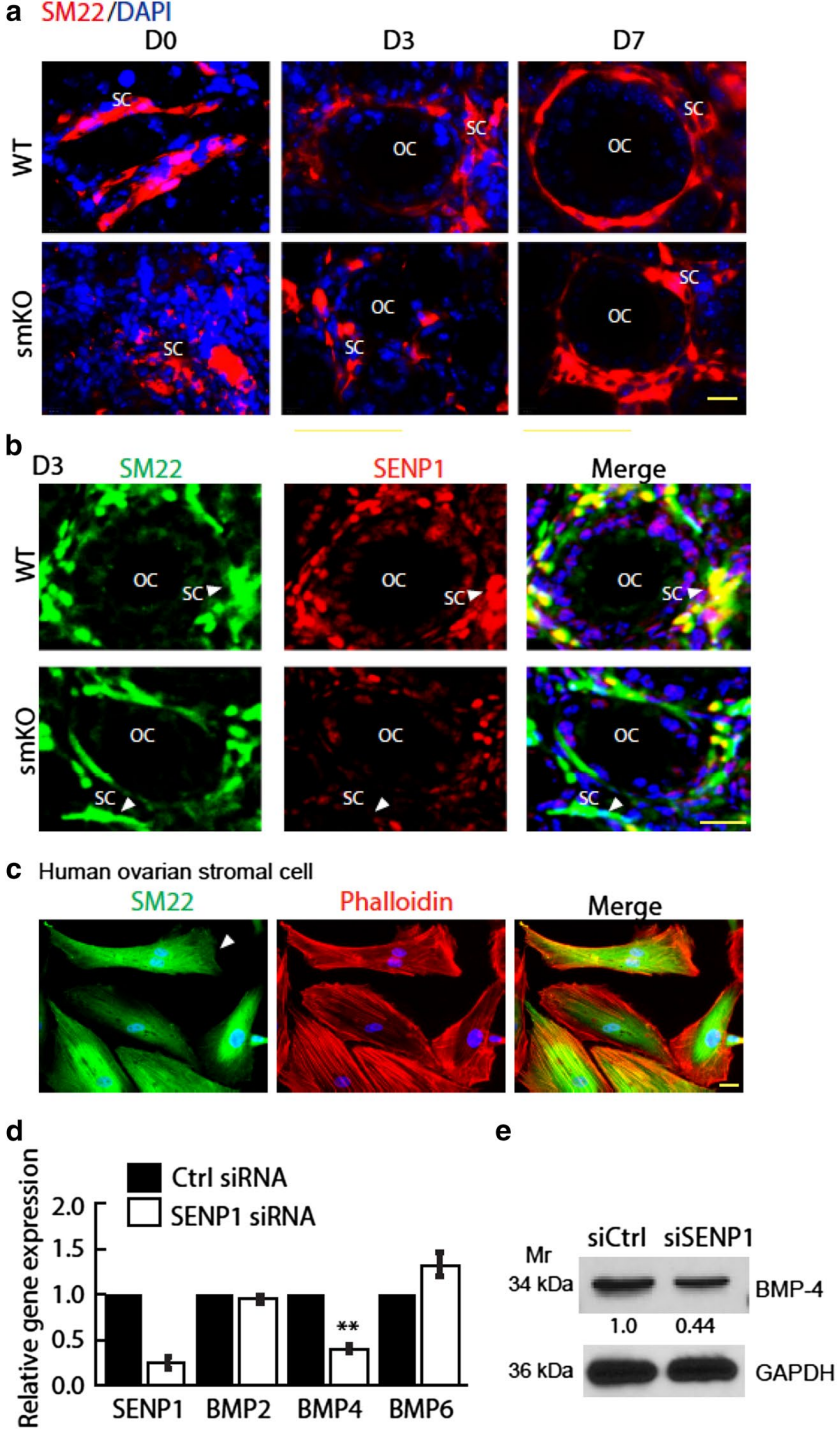
SENP1-deficient stromal cells are disorganized with reduced expression of BMP4. **a** SENP1-deficient stromal cells are disorganized. Stromal cell marker SM22α immunofluorescence in ovaries of WT and SENP1-smKO mice at D0, D3 and D7. SM22α^+^ stromal cells gradually mature to tightly wrap advanced follicles In WT ovaries. However, SENP1-smKO stromal cells exhibit delayed maturation and loosely surround follicles. *Scale bars* 15 μm. *OC* oocyte, *SC* stromal cell. **b** SENP1 and SM22α co-immunofluorescence staining of ovaries from WT and SENP-smKO mice. **c** Immunofluorescence staining of sm22α and phalloidin of human primary ovarian stromal cells. *Scale bars* 10 μm. **d**, **e** SM22α^+^ stromal cells were transfected with control siRNA or SENP1 siRNA. **d** SENP1, BMP2, BMP4 and BMP6 mRNAs were determined by qRT-PCR. Normalized mRNA levels (with GADPH) are presented as fold changes by taking control siRNA group as 1.0. **e** Protein level of BMP4 was determined by western blot with respective antibodies. Normalized protein levels (with GADPH) are presented as fold changes by taking control siRNA group as 1.0. Three independent experiments were performed

To further detect changes of stromal cells in SENP1-smKO mice in which a delayed oocytes growth and decreased follicles formation were observed in early ovarian development, ovaries from WT and SENP1-smKO mice stained with anti-SM22α antibody, we detected well-distributed SM22α expression in stromal cells surrounding the follicles of WT ovaries. In contrast, un-uniform distributions of SM22α stromal cells were observed in SENP1-smKO ovaries at both D0 and D3 (Fig. 4a). SENP1 deletion in SM22α^+^ cell was confirmed by co-immunostaining with anti-SENP1 and anti-SM22α (Fig. 4b). BMPs have been identified important roles during formation, growth and maturation of ovarian follicles in mammals [27–32]. Among them, BMP4 derived from stromal/somatic cells is critical in promoting the survival and development of primordial follicles in the neonatal ovary, especially at the primordial-to-primary follicle transition. To investigate if SENP1 regulates gene expression of stromal BMPs, we carried out a series of experiments using the primary human ovarian stromal cells (from Department of Obstetrics and Gynecology, Yale School of Medicine), which proved to be SM22α positive (Fig. 4c). We determined effects of SENP1 knockdown on BMPs expression in stromal cells by qRT-PCR. BMP4, but not other BMPs, was significantly reduced by SENP1 siRNA silencing (Fig. 4d). Expression of BMP4 protein level was also significantly decreased in SENP1-knockdown stromal cells (Fig. 4e). These results suggest that SENP1 is critical for BMP4 expression in stromal cells.

### Stromal SENP1 deletion reduces expression of BMP4 receptor BMPR1B and BMPR2 on oocytes

**Fig. 5:**
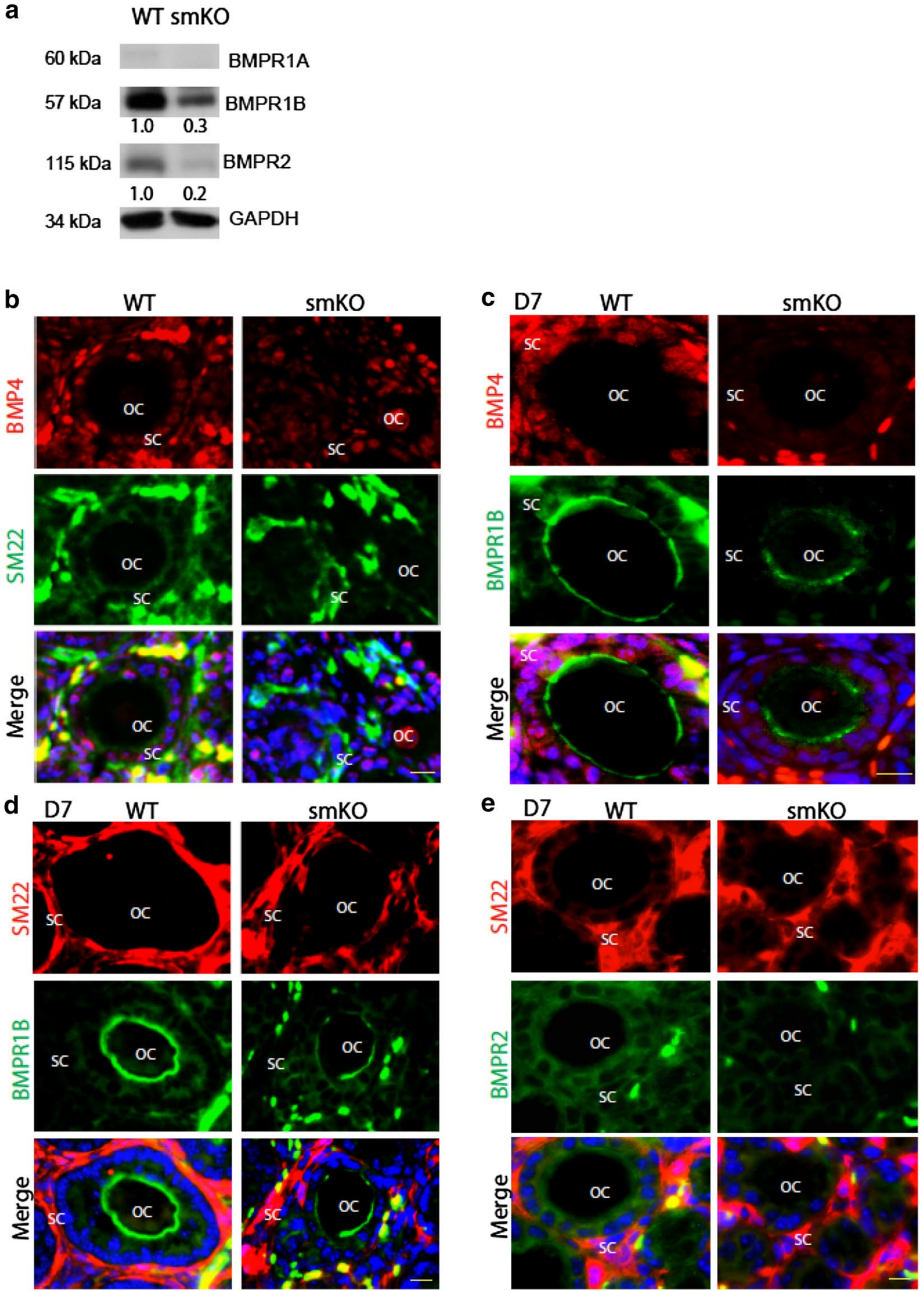
BMPR1b expression in developing oocytes was reduced in SENP1-smKO mice. Ovaries from WT and SENP-smKO mice were harvested at postnatal day 7. **a** Protein levels of BMP4 and receptors were determined by western blot with respective antibodies. Normalized protein levels (with GADPH) are presented as fold changes by taking WT group as 1.0. n = 3. **b** BMP4 and SM22α co-immunofluorescence staining of ovaries from WT and SENP-smKO mice. **c** BMP4 and BMPR2 co-immunofluorescence staining of ovaries from WT and SENP-smKO mice. **d**, **e** SM22α and BMPR1B or BMPR2 co-immunofluorescence staining of ovaries from WT and SENP-smKO mice at postnatal day 7. *Scale bars* 15 μm. *OV* ovary, *OC* oocyte, *GC* granulosa cell, *SC* stromal cell. *Asterisks* indicate the same oocyte in the merged panel

To further investigate potential involvement and relative functions of stromal SENP1 in regulating early stage of oocytes and follicles development, we further examined expressions of BMP4 and its cognate receptors BMPR1A, BMPR1B and BMPR2 in vivo. Consistent with previous reports ref, BMPR1B and BMPR2, but not BMPR1A, were highly expressed in developing ovaries (Fig. 5a). To determine which cell type contributes to BMPR expression, we performed co-immunostaining for BMP4 and its receptors. BMP4 was primarily expressed by SM22α stromal cells surrounding follicles in WT ovaries. Consistent with the in vitro findings, BMP4 was greatly reduced in the stromal cells of SENP1-smKO ovaries (Fig. 5b). As previously reported, BMPR1B and BMPR2 were detected on oocytes (Fig. 5c–e). Interestingly, we observed reduced expression of BMPR1B (Fig. 5c, d) and BMPR2 (Fig. 5e) in oocytes of SENP1smKO mice, consistent with a delayed maturation of oocytes in these mice.

## Discussion

Folliculogenesis is a complex process that depends on numerous factors including both extra-ovarian and intra-ovarian factors. It has long been recognized that oogenesis and folliculogenesis require complex bidirectional signaling between the oocyte and the surrounding stromal/somatic cells; while stromal cells support oocyte development, oocytes promote surrounding stromal cells differentiation and proliferation [10–14]. Howev-er, the molecular mechanism underlying the interactions between oocyte and stroma remains unknown. Our study supports that SENP1 in ovarian stroma is crucial for maintenance and survival of folliculogenesis. It is evident that mice with a deletion of the SENP1 gene in SM22^+^ ovarian stromal cells (SNEP1-smKO) exhibit an incomplete breakdown of germ cell cysts with reduced number of primary and secondary follicles on both postnatal day 3 and day 7. Consequently, the number of total follicles in adolescent and adulthood of SENP1-smKO mice maintain fewer than wild-type littermates, leading to premature ovarian failure in old (>8 months of age) SENP1-smKO mice. In humans, premature ovarian failure, also known as premature ovarian insufficiency (POI) or primary ovarian insufficiency, is the loss of function of the ovaries before age 40 [10]. Our SENP1-smKO mice may provide a useful mouse model for human POI to investigate the pathogenesis and underlying mechanism for POI. The distribution of SM22^+^ ovarian stromal cells surrounding the follicles in SENP1-smKO mice was drastically disrupted at early stage, suggesting that alterations of cellular organization of follicle contribute to defective development process.

**Fig. 6:**
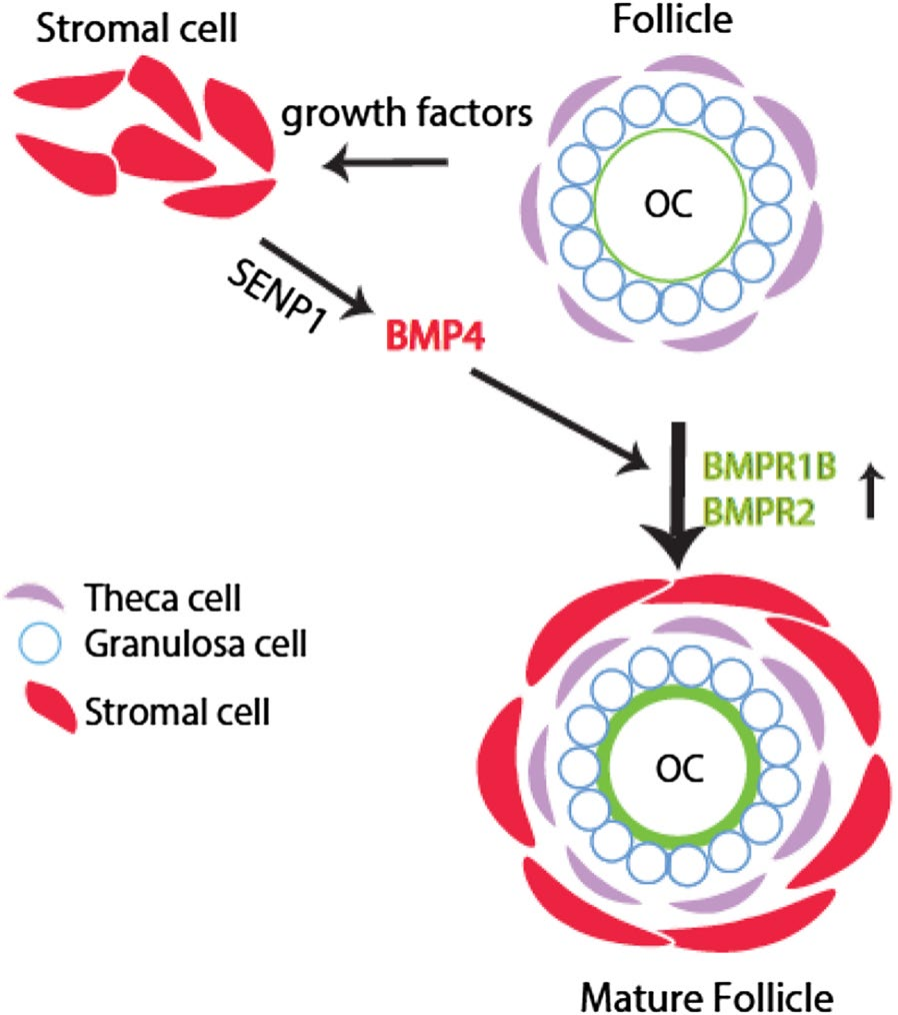
A model for the role of SENP1 in early folliculogenesis. Mammalian oocytes develop within ovarian follicles. It is known that oocyte/theca/granulosa cells secret growth factors (e.g., FGF2) to stimulate stromal cell maturation, and stromal cell-derived BMPs (such as BMP4) facilitate follicle maturation. Our current data support that SUMO endopeptidase SENP1 is critical for BMP4 expression in stromal cells, which in turn upregulates BMPR1B and BMPR2 on oocytes during early follicle development. Stromal SENP1 deletion alters stromal cell morphology and maturation as well as BMP4 expression, leading to delayed folliculogenesis

In mammals, the growth of oocytes and development of follicles within the ovary are highly dependent on autocrine and paracrine growth factors from granulosa cells, theca cells, stromal interstitial cells, and the oocytes [27–32]. The bone morphogenetic protein (BMP) family belongs to the transforming growth factor (TGF-β) superfamily. BMPs bind to type I and type II serine–threonine kinase receptors, and transduce signals through the SMAD signaling pathway. Among BMP proteins, BMP4 and BMP-7 are reported to be of stromal cell and/or pre-thecal origin [27–32]. BMP4 and its receptors BMPR-IB and BMPR-II (and BMPR-IA, weakly) have been detected in mouse ovaries [27, 28, 30–32]. Our data indicate that SENP1 deficiency in SM22^+^ mouse stromal cells, effectively reduces BMP4 mRNA and protein expression in ovary. Knockdown of SENP1 in SM22^+^ human ovarian stromal cells also effectively attenuates expression of BMP4, but not BMP2 or BMP6. It has been experimentally confirmed that BMP4 promotes primordial follicle development and the primordial-to-primary follicle transition. Mice treated with BMP4 have significantly higher proportion of developing primary follicles and fewer arrested primordial follicles than untreated controls. Conversely, mice treated with neutralizing antibody against BMP4 have markedly smaller ovaries, associating with a progressive loss of oocytes and primordial follicles, accompanying loss of normal ovarian tissue morphology over time. More interestingly, BMP4 protein is localized in stromal cell populations associated with developing primordial follicles [29]. Taken together, SENP1, by regulating BMP4 expression in stromal cells concomitant with decreased expression of BMP4 receptor BMPR1b and BMPR2 on oocytes, promotes ovarian maturation (Fig. 6).

Regulation of BMP4 gene expression has been investigated non-ovarian stromal cells and results suggest that BMP4 expression is regulated by several transcriptional factors. Transcriptional factor SOX2 could negatively regulate BMP4 promoter activity, possibly through binding to the promoter located in the first intron region of BMP4. Interestingly, SOX2 can be SUMOylated at the lysine 247 and this modification inhibits the DNA binding of SOX2 [33]. SOX2 is a member of the high mobility group (HMG) domain DNA-binding proteins for transcriptional control and chromatin architecture. The HMG domain of SOX2 binds the DNA to facilitate transactivation by the cooperative transcription factors such as OCT3/4. Therefore, SOX2 together OCT3/4 regulate many critical genes involved in stem cell marker genes and developmental genes. Similar to SOX2, OCT3/4 are also regulated by SUMOylation [34]. The BMP4 gene promoter also contains an AP-1 element therefore BMP4 expression can be regulated by transcriptional factor AP-1, and the integrin receptor, ILK, p38, and JNK signaling pathways [35]. It is well documented that AP-1 and upstream signaling are regulated by SUMOylation. We will investigate if SENP1, by modulating SUMOylation of SOX2-OCT1/4 or AP-1, regulate BMP4 expression in the ovarian stromal cells. BMP4 expression is also regulated at mRNA levels. LincRNA MEG3, via suppressing SOX2, positively regulates BMP4 transcription. Specifically, MEG3 could dissociate the transcription factor SOX2 from the BMP4 promoter [36]. Lin28, a stem cell factor, binds to BMP4 mRNA, thereby promoting BMP4 expression at the post-transcriptional level [37]. It has not been explored if LncRNA MEG3 and Lin28 are regulated by SUMOylation. Taken together, our study warrantee further investigation to define the mechanisms by which SENP1-SUMO mediates BMP4 gene expression, which will provide potential therapeutic targets for human POI and other ovarian associated diseases such as ovarian cancer.

## Conclusions

The mechanism by which stromal cells and derived factors regulate oocyte development remains unclear. Our present study has revealed that protein SUMOylation-regulating enzyme SENP1 plays a critical role in early ovarian follicle development by regulating gene expression of BMP4 in stroma and stroma-oocyte communication.

## Methods

### Smooth muscle 22α(SM22α) specific SENP1 knockout mice

SENP1^+/lox^ mice were generated by inserting loxP sites surrounding the SENP1 gene exons 5 and 6, based on homologous recombination 17. SENP1 lox/lox mice were obtained by intercrossing SENP1^+/lox^ mice. SENP1^lox/lox^ mice were mated with three different deleter lines carrying the Cre recombinase driven by the SM22α (obtained from Jackson Laboratory). All mice had been subsequently backcrossed onto the C57BL/6 background for 46th generations. The deletion of SENP1 in uterine stromal cells of SENP1^lox/lox^: Cre was verified by quantitative PCR with reverse transcription using primers amplifying exons 5–6 [19, 38] and SENP1^+/+^ and specific Cre or SENP1^lox/lox^ mice used as controls. Mice were cared for in accordance with National Institutes of Health guidelines, and all procedures. All animal studies were approved by the Institutional Animal Care and Use Committee of Yale University.

### Immunofluorescence staining

Antibodies used for immunofluorescent staining. Confocal microscopy images were taken with a Zeiss-LSM 700 microscope and evaluated using the ZEN2010 software. For mean fluorescence intensity measurements, confocal microscopy images were analyzed with ImageJ. Slides were observed using a Zeiss Axiovert 200 fluorescence microscope (Carl Zeiss MicroImaging; Thornwood, NY), and images were captured using Openlab3 software (Improvision, Lexington, MA). For tissue, 5 μm serial sections cut from frozen, OCT-embedded tissues were fixed in −20 °C acetone for 10 min, dried for 15 min, followed by the same blocking/antibody protocol for cells as listed above.

### Cell culture and RNA interference for SENP1

Human ovarian stromal cells (HOSC) were obtained from Department of Obstetrics and Gynecology, Yale School of Medicine and grown in DMEM media supplemented with 2 mM glutamine and 15% FBS. In view of the established characteristics of siRNA- targeting constructs, we designed three pairs of siRNA oligonucleotides for SENP1: siRNA 21696: 5-GGAAAUGGAGAAAGAAAUA dTdT-3; siRNA21512: 5-GGA CCAGCUUUCGCUUUCU dTdT-3; siRNA21605: 5-GGACAUUUGGACCGA UCUU dTdT-3. Three siRNAs were obtained similar knockdown efficiency. The corresponding scramble siRNA oligonucleotide for siRNA21512: 5-GGA CCA GCA UAC GCU UUCU dTdT-3, with two nucleotide mutations (underlined), was synthesized from Ambion (Austin, TX, USA). For each transient transfection, siRNAs (10 μM) and normalized plasmid (10 mM)were transfected into cells by Oligofectamine (Life Technologies, Inc.; Invitrogen), according to the manufacturer’s instructions (Invitrogen). Cells were cultured for 48 or 72 h before harvest.

### Quantitative PCR (qRT-PCR)

Total RNA was extracted from human tissues using the RNeasy Plus Mini Kit (74134, Qiagen), and then converted into cDNAs using the High Capacity cDNA Reverse Transcription Kit (4368814, Applied Biosystems) following the manufacturer’s instruction. Quantitative PCR was performed with a CFX-96 (Bio-Rad) using the RT2 SYBR Green (330500, SA Biosciences). All values were normalized with GAPDH abundance. Data were presented as the average of triplicates ± SD.

### Western blot

Murine and human primary myometrium cells were directly lysed in Laemmli sample buffer (Bio-Rad) containing β-mercaptoethanol. Lysates were resolved on Bio-Rad precast gradient gels (4–20%) and transferred onto nitrocellulose membranes. After blocking (5% non-fat dried milk in Tris buffered saline (TBS) with 0.1% Tween-20), membranes were probed with antibodies (1:200) for BMP4 (ab39973), BMPR1A (ab38560), BMPR1B (ab78417) and BMPR2 (ab106266), using anti-GAPDH (1:2000; Cell signaling) as a loading control, overnight at 4°.

### Statistical analysis

The differences of results of oocytes counting, western-blot and qRT-PCR were analyzed by student t test. Statistical analyses in this study were performed using SAS software (version 9.1.4, SAS Institute, Cary, NC). All statistical tests were two-tailed, and *P* values less than 0.05 were considered statistically significant.

## Additional file

**Additional file 1: Figure S1.** Endothelial cell-specific deletion of SENP1 has no effects on the size or number of developing follicles. (**A**, **B**) Hematoxylin and Eosin staining of ovaries from WT and SENP1-ecKO mice at postnatal day 3 and day 7. Scale bars: 160 μm. Follicle size in ovaries of WT and SENP1-ecKO mice are quantified. Data are presented as means ± SEM, n = 5. (**C**) Number of total follicle in ovaries of WT and SENP1-smKO mice at ages of 3, 6 weeks and 8 months were quantified based on H&E staining. Data are presented as means ± SEM, n = 5.
