# Supplementary material for: Stromal Senp1 promotes mouse early folliculogenesis by regulating BMP4 expression
Source: Cell Biosci. 2017 Jul 25;7:36. doi: 10.1186/s13578-017-0163-5 (PMC5526272; doi:10.1186/s13578-017-0163-5)
Supplement: Supplementary file 1 — Additional file 1: Figure S1. Endothelial cell-specific deletion of SENP1 has no effects on the size or number of developing follicles. (A, B) Hematoxylin and Eosin staining of ovaries from WT and SENP1-ecKO mice at postnatal day 3 and day 7. Scale bars: 160 μm. Follicle size in ovaries of WT and SENP1-ecKO mice are quantified. Data are presented as means ± SEM, n = 5. (C) Number of total follicle in ovaries of WT and SENP1-smKO mice at ages of 3, 6 weeks and 8 months were quantified based on H&E staining. Data are presented as means ± SEM, n = 5. [file 13578_2017_163_MOESM1_ESM.pdf]

SUPPLEMENTAL DATA

Stromal Senp1 promotes mouse early folliculogenesis by regulating BMP4 expression

Shu Tan et al

Tan et al Supplemental Fig.S1

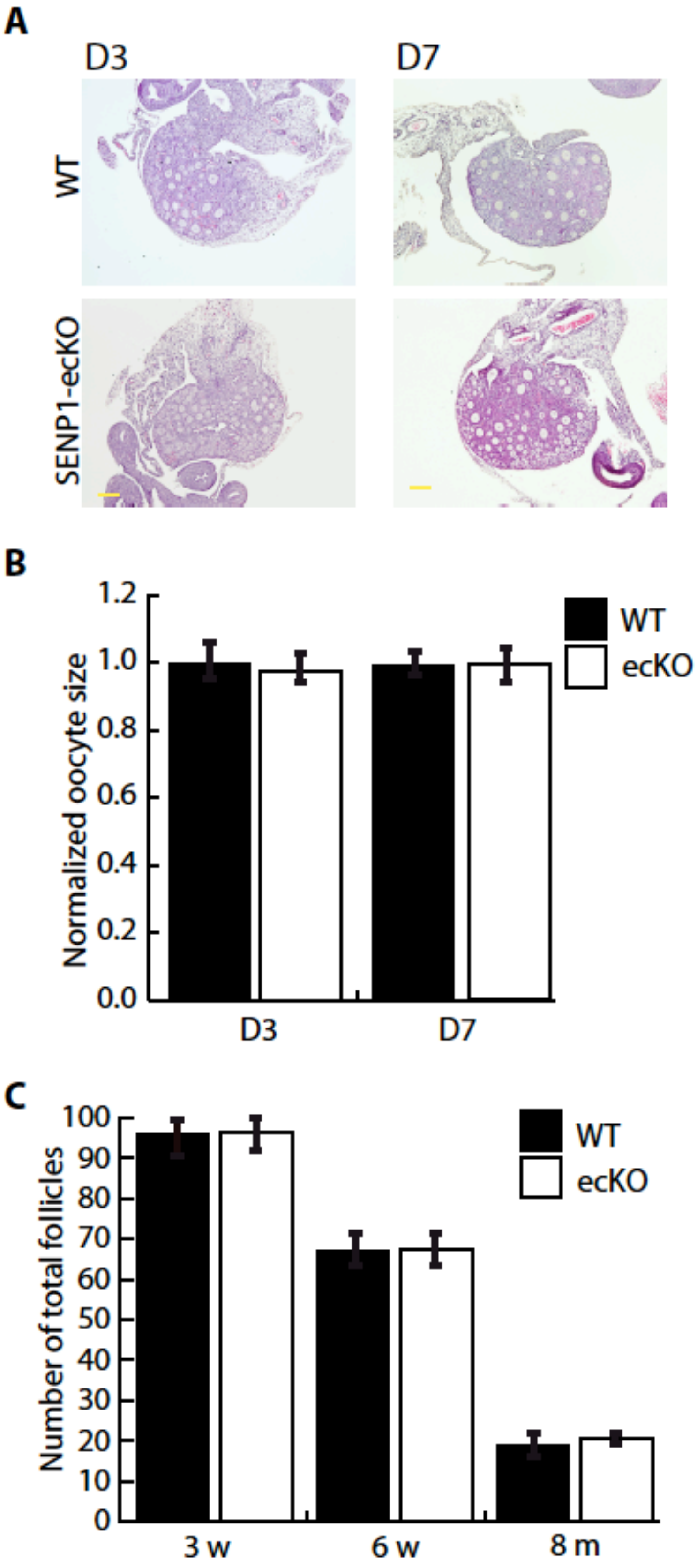

Supplemental Fig.S1. Endothelial cell-specific deletion of SENP1 has no effects on the size or number of developing follicles.

(**A-B**) Hematoxylin and Eosin staining of ovaries from WT and SENP1-ecKO mice at postnatal day 3 and day 7. Scale bars: 160 μm. Follicle size in ovaries of WT and SENP1-ecKO mice are quantified. Data are presented as means ± SEM, n=5. (**C**) Number of total follicle in ovaries of WT and SENP1-smKO mice at ages of 3 weeks, 6 weeks and 8 months were quantified based on H&E staining. Data are presented as means ± SEM, n=5.
